# Trait Cheerfulness Does Not Influence Switching Costs But Modulates Preparation and Repetition Effects in a Task-Switching Paradigm

**DOI:** 10.3389/fpsyg.2017.01013

**Published:** 2017-06-22

**Authors:** Raúl López-Benítez, Hugo Carretero-Dios, Alberto Acosta, Juan Lupiáñez

**Affiliations:** ^1^Department of Experimental Psychology, Mind, Brain and Behavior Research Center, Faculty of Psychology, University of GranadaGranada, Spain; ^2^Department of Methodology of Behavioral Sciences, Mind, Brain and Behavior Research Center, Faculty of Psychology, University of GranadaGranada, Spain

**Keywords:** sense of humor, trait cheerfulness, task switching, cognitive flexibility, attribute repetition, preparation

## Abstract

Many studies have shown the beneficial effect of positive emotions on various cognitive processes, such as creativity and cognitive flexibility. Cheerfulness, understood as an affective predisposition to sense of humor, has been associated with positive emotions. So far, however, no studies have shown the relevance of this dimension in cognitive flexibility processes. The aim of this research was to analyze the relationship between cheerfulness and these processes. To this end, we carried out two studies using a task-switching paradigm. Study 1 aimed at analyzing whether high trait cheerfulness was related to better cognitive flexibility (as measured by reduced task-switching costs), whereas Study 2 aimed at replicating the pattern of data observed in Study 1. The total sample was composed of 139 participants (of which 86 were women) selected according to their high versus low scores in trait cheerfulness. In a random way, participants had to judge whether the face presented to them in each trial was that of a man or a woman (gender recognition task) or whether it expressed anger or happiness (expressed emotion recognition task). We expected participants with high versus low trait cheerfulness to show a lower task-switching cost (i.e., higher cognitive flexibility). Results did not confirm this hypothesis. However, in both studies, participants with high versus low trait cheerfulness showed a higher facilitation effect when the stimuli attributes were repeated and also when a cue was presented anticipating the demand to perform. We discuss the relevance of these results for a better understanding of cheerfulness.

## Introduction

Nowadays, one of the main areas of interest in the sense of humor field has been to provide a global theoretical framework to guide research. In this sense, Ruch et al. (1996, 1997) developed a theoretical model focused on isolating the temperamental basis of sense of humor: cheerfulness, seriousness, and bad mood.

Cheerfulness, the subject of this research, is understood as a predisposition to smile/laugh and express positive emotions in response to humorous stimuli, alongside a general tendency to show a positive and a joy affective state. This dimension comprises five facets: the prevalence of a cheerful mood, a low threshold for smiling and laughter, a composed view of adverse life circumstances, a broad range of active elicitors of cheerfulness and smiling/laughter, and a generally cheerful interaction style. In the model (Ruch and Hofmann, 2012) only cheerfulness encourages hilarity^1^.

Ruch and colleagues developed an inventory to assess the individual differences and connections that may exist between the affective and cognitive basis laid out in the model from both a trait perspective [State-Trait Cheerfulness Inventory-Trait Version (STCI-T); Ruch et al., 1996] and a state perspective [State-Trait Cheerfulness Inventory-State Version (STCI-S); Ruch et al., 1997]. This fact, along with the extensive body of knowledge obtained on cheerfulness over the last 20 years, has contributed to its development from both a theoretical and empirical point of view.

Previous research has shown that cheerfulness plays an important role in humor. In this sense, it has been pointed out that cheerfulness affects dispositions of the exhilaration response (Ruch, 1997), predicts most of sense-of-humor facets, contributes to the use of humor as a recovery strategy, and is associated with affiliative and self-enhancing humor styles (Ruch and Hofmann, 2012). Moreover, other research support the applicability and relevance of cheerfulness in areas as diverse as personality, health, or emotion (e.g., Ruch et al., 1996, 1997; Yip and Martin, 2006; Ruch and Köhler, 2007; Papousek and Schulter, 2010; Carretero-Dios et al., 2011; Ruch and Hofmann, 2012; Delgado-Domínguez et al., 2016).

Thus, the concept of cheerfulness can be granted similar virtues to those attributed to positive emotions (see Lyubomirsky et al., 2005, for a review). For instance, it has been established that trait cheerfulness is closely associated with better physical and psychological well-being, an increased manifestation and expression of positive emotions, satisfaction, and quality of life, better resilience, ability to cope, and recovery from stressful situations, a greater ability to use creative thinking, and high interpersonal skills (Papousek and Schulter, 2010; Ruch and Hofmann, 2012).

Within the area of research on positive emotions, several studies have highlighted the influence of such emotions on cognitive flexibility (e.g., Wadlinger and Isaacowitz, 2006). The results obtained can be included in Fredrickson’s (2001) broaden-and-build theory, which suggests that positive emotions expand our mental and behavioral repertoire. As a consequence, after being exposed to positive affective states our scope of attention broadens (see, for example, Johnson et al., 2010) and aspects of cognition such as cognitive flexibility increase, leading to an adaptation to changes in the environment. In this regard, it should be noted that the conceptualization of cheerfulness as a positive affective dimension linked to sense of humor leads us to wonder how relevant this factor is for the study of cognitive flexibility.

### Cognitive Flexibility and Control Processes

Control processes are related to individuals’ ability to select relevant information and ignore irrelevant information when performing a task (Posner and Rothbart, 2007). They are also related to cognitive flexibility (Davidson et al., 2006), understood as the ability to modify one’s way of thinking or acting in accordance with changing demands.

Some authors argue that cognitive control has three central components: the inhibition of whatever is irrelevant to the fulfillment of our goals, the updating and monitoring of the information, and the switch between mindsets to activate the relevant material for the particular demand at hand (Miyake et al., 2000). When we perform two or more tasks alternately, we must constantly reconfigure our mindset to respond to the new demand (Crone et al., 2006). The ease with which these readjustments are carried out is the key defining characteristic of cognitive flexibility, which is a fruitful process for adapting to the environment.

Studies on control processes and cognitive flexibility have used numerous tasks (e.g., Stroop, 1935; Simon, 1969; Eriksen and Eriksen, 1974). Recently, one of the most widely used experimental procedures to explore cognitive flexibility has been task switching (Monsell, 2003; Kiesel et al., 2010). In task-switching tasks, participants are instructed to perform one of two possible tasks in each trial. In some consecutive trials the same demand is repeated, while in others it is different. This makes it possible to determine the task-switching cost, measured as the difference in performance when the task changes in two consecutive trials, compared to when it is repeated.

It has additionally been proved that, in this type of task, the amount of stimuli attributes that either repeats or changes on consecutive trials can also affect behavior and the typical effects of task switching costs. When an individual is exposed to a stimulus, a mental file is created about this event, including the attributes of the stimulus as well as the response to it. This representation is subsequently reactivated in the presence of similar stimuli, thus affecting the performance of tasks involving these stimuli (Hommel, 2004). In this regard, it has been reported that total attribute repetition only has a beneficial effect if the response is the same in two consecutive trials (Kahneman et al., 1992). However, the performance is worse when there is partial attribute repetition than when there is no attribute repetition (or when all the attributes are repeated). This is because, although in some cases this repetition may help solve the demand, it normally requires reconfiguring the previously created mental file (Hommel, 1998, 2004). Additionally, some studies have included cognitive or affective demands, or between two different cognitive demands in the presence of the same stimuli, which have made it possible to determine the task-switching cost between two consecutive trials depending on the type of demand (e.g., Egner et al., 2008; Ochsner et al., 2009; Schuch et al., 2012). Importantly, both the repetition of attributes and the type of task interact with task switching (Marzecová et al., 2013) and therefore should be considered when studying task-switching costs.

Despite the lack of any existing literature on the modulation of cognitive flexibility processes by cheerfulness, some studies are beginning to offer clues on their possible relationship. Previous research has pointed out that the induction of positive affective states, which are related to cheerfulness, are associated with a better cognitive flexibility (Baumann and Kuhl, 2005; Yang and Yang, 2014). From a correlational perspective, it has been established that cheerfulness is linked to some personality variables of interest for the current research (Ruch and Köhler, 2007). For example, Carretero-Dios et al. (2014) observed positive relationships among trait cheerfulness, extraversion, openness, and agreeableness, and negative relationships between trait cheerfulness and neuroticism. And, importantly, some studies have found that such personality characteristics may modulate performance on tasks that requires cognitive flexibility (Murdock et al., 2013). For example, while positive associations among openness (DeYoung et al., 2005), agreeableness (Jensen-Campbell et al., 2002), and cognitive flexibility has been observed, extraversion (Campbell et al., 2011) and neuroticism (Compton, 2000) seem to contribute to reduce it.

Links between cognitive flexibility and sense of humor could also contribute to explain the possible modulation by cheerfulness. Some studies derived from clinical populations, such as Asperger’s syndrome (Weiss et al., 2013) or Schizophrenia (Tsoi et al., 2008; Polimeni et al., 2010) have found reduced sensitivity to recognize or discriminate humor in these populations, perhaps reflecting a deficit in cognitive functions such as cognitive switching. In fact, one important component of humor response has been related to cognitive processes related to re-interpretation of evidence and congruity resolution, which involves cognitive flexibility (Suls, 1972). Furthermore, it has been established that cognitive flexibility and the use of emotion regulation strategies are positively related (e.g., Gul and Khan, 2014). For example, Malooly et al. (2013) found that a lower task-switching cost predicted the success to use reappraisal strategies to down-regulate negative emotions.

More specifically, cheerfulness has been specifically associated to cognitive flexibility; in its third facet -composed view of adverse life circumstances- it is assumed that high trait cheerfulness individuals are good in re-interpreting events (e.g., “Most problems turn out to be not as bad as all that when considered calmly and composedly”). Therefore, given that trait cheerfulness is an important key to understand and produce humor, and trait cheerfulness has been associated to a high ability to cope with negative events (Ruch and Hofmann, 2012), people scoring high in trait cheerfulness might also have better executive functioning.

To test this hypothesis, we conducted a study in our laboratory (López-Benítez et al., unpublished) in which participants differentiated in trait cheerfulness (assessed with the STCI-T) were required to perform the following task-switching paradigm: in a random way, in each trial, they had to say whether the face presented to them on a screen was that of a man or a woman (gender recognition task) or if the face expressed anger or happiness (expressed emotion recognition task). The task could change, or not, between two consecutive trials. The various conditions of repetition of the stimuli attributes were also analyzed (Kahneman et al., 1992; Hommel, 1998, 2004). With the additional goal of studying interference effects, the faces were always presented with a written word at the center that could match their gender or expression (congruent trials) or not (incongruent trials) (depending on the task; e.g., Etkin et al., 2006). Results showed an interesting trend: individuals with high trait cheerfulness showed a lower task-switching cost than those with low trait cheerfulness, especially in the conditions in which all the attributes were repeated between consecutive trials. These results were interpreted as showing that these individuals have higher cognitive flexibility in repetition conditions, precisely where cognitive flexibility is most necessary.

However, this interpretation should be taken cautiously due to several factors. First, the size of the observed effect was small (0.05) and the interaction between task change, group, and attribute repetition was only marginally significant, all of which suggests that the result should be further studied. Moreover, in that study we included the interference variable. Although this variable did not interact with trait cheerfulness, it might affect the analysis of task-switching costs as participants had to use more cognitive resources, especially on incongruent trials, which made the task especially harder.

In spite of the relevance of studying cheerfulness and cognitive flexibility, there are still no studies that have deepened on their possible relationships. In this study, we aimed at bridging this gap. As a first step and from a systematic point of view, we wanted to analyze whether trait cheerfulness had an impact on cognitive flexibility processes. We consider that this study is highly relevant because if cheerfulness indeed plays a role on executive functions such as cognitive flexibility, the assessment and training of cheerfulness could be considered as a relevant aspect in the improvement of skills focused in adaptation to the environment, which is a basic human function. In addition, with this study we could check whether previous relationships between cognitive processes and humor are expanded to a predisposition (as a trait) to sense of humor at the same time that its temperamental basis theoretical model is empirically tested (Ruch et al., 1996).

To achieve that aim, two studies were carried out. In both studies, two groups of participants scoring high versus low in trait cheerfulness performed a task-switching paradigm. In Study 1, we analyzed whether high trait cheerfulness people had better cognitive flexibility (as measured by a lower task-switching cost), whereas in Study 2 we extended and checked the consistency of the pattern of data observed in the first study.

## Study 1

Taking previous research into account, we conducted this study to analyze whether trait cheerfulness (operationalized with the STCI-T) could be directly related to cognitive flexibility through a task-switching paradigm. To this end, as in our previous unpublished study (above described), participants carried out a task in which they had to correctly identify either the emotion or the gender of a face presented in the center of the screen; this task was randomly repeated or alternated between consecutive trials. However, in this study, in order to simplify the experimental design, we removed the interference variable, that is, we did not present a word superimposed on the faces. In addition, half of the trials were preceded by a cue that anticipated the upcoming task, allowing participants to get ready for it. The inclusion of this variable is important, as it has been proven that the presentation of a cue that anticipates the demand reduces the cognitive effort required, which is likely to lead to a better performance in this type of task (see Kiesel et al., 2010). Based on the above-mentioned studies and taking into account that several studies have shown that positive affective states are associated with a lower task-switching cost (Yang and Yang, 2014), we predicted that, compared to individuals with low trait cheerfulness, individuals with high trait cheerfulness would have greater cognitive flexibility, thus showing a lower task-switching cost, particularly when performing trials that require greater cognitive flexibility (i.e., attribute repetition and no prior preparation).

### Material and Methods

#### Participants

The sample was composed of 49 students from the University of Granada, who were selected from a total of 244 people according to their high versus low trait cheerfulness scores, obtained with the Spanish version of the STCI-T, cheerfulness dimension (Carretero-Dios et al., 2014). The average score ± 1 SD was used as a criterion to create the groups. Specifically, the high trait cheerfulness group comprised 24 participants (20 women, mean age 19.50 years, *SD* = 5.82, cut-off score ≥ 3.42), and the low trait cheerfulness group was made up of 25 participants (20 women, mean age 21.60 years, *SD* = 7.65, cut-off score ≤ 2.68). All participants had normal or corrected-to-normal vision, participated in the study voluntarily, and received course credit in exchange for participating. They signed an informed consent and had the possibility to stop the experimental session without any consequences. Data from one participant were not taken into account because the number of correct responses was low compared to the group (more than 2.5 SD below from the group mean). The study was part of a broader research project, approved by the Ethics Committee of the University of Granada, in accordance with the 1964 Declaration of Helsinki.

#### Stimuli

In order to conduct the study, eight photographs were selected from the database of the Karolinska Institute in Stockholm, Sweden (Lundqvist et al., 1998; Goeleven et al., 2008). The images showed two happy men (AM25HAS; AM10HAS), two angry men (AM09ANS; AM02ANS), two happy women (AF31HAS; AF14HAS), and two angry women (AF20ANS; AF25ANS). All the photographs were 141 mm × 191 mm in size. Additionally, a 100 ms sound was used to provide participants feedback on their performance during the practical part of the experiment.

#### Procedure

Participants went to the laboratory individually and were led to a soundproofed, dimly lit room. They were seated in a comfortable chair in front of a 15-inch computer monitor, at a distance of 60 cm. They gave their consent prior to the start of the experiment. Next, the researcher informed them that the goal of the study was to analyze their performance in a psychological task, to which they should respond as quickly as possible while trying to avoid any errors.

The researcher explained how they should respond to the task, and was present during some practice trials to ensure that they were performing them correctly. After that, the researcher left the room and the experimental trials were presented.

At the beginning of each trial, a fixation point appeared in the center of the screen for 1 s. Randomly, in half of the trials a green or purple mark (preparation condition) also appeared around the fixation point, anticipating the task participants had to perform next. After the second, one of the eight photographs previously described appeared on the screen, surrounded by a green or purple frame, which indicated the nature of the task to perform: to indicate either the emotion on the face (happiness vs. anger) or the gender (man vs. woman). In the half of the trials in which the colored frame did not appear along with the fixation point (no preparation condition), the frame was presented simultaneously with the photograph. The different trials were presented randomly and the specific sequences of consecutive trials were coded off-line in order to code the other variables of interest. Thus, in approximately half of the trials, the task was the same in two consecutive trials (same task), while in the rest of the trials it changed (different task). On the other hand, sometimes the attributes of the stimuli (gender and emotion) were repeated in two consecutive trials (complete repetition), whereas in other cases these characteristics were not repeated at all (complete alternation) or only one of them was repeated (partial repetition).

**FIGURE 1:**
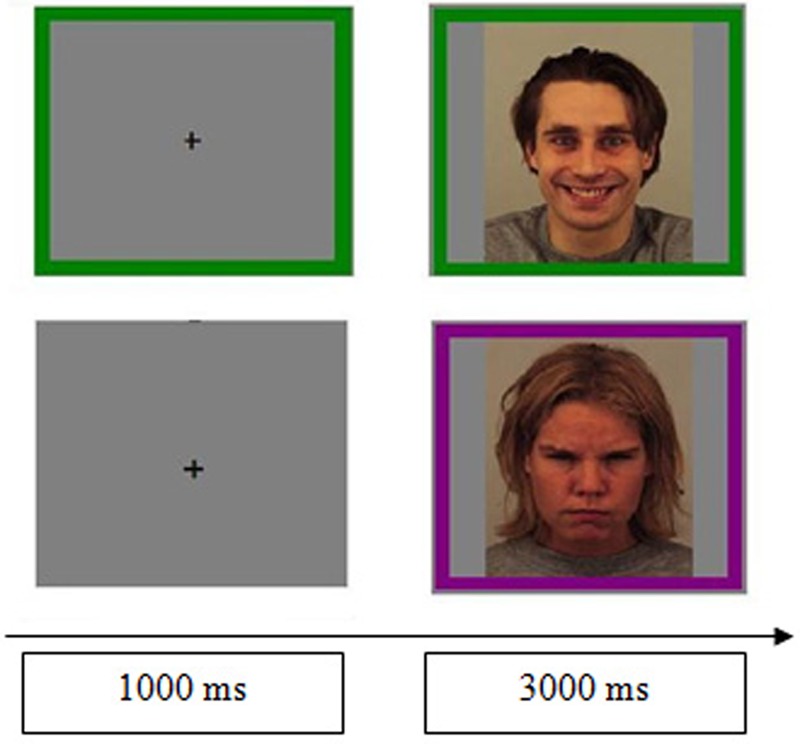
Sequence of events in two trials. In the upper example, which illustrates the preparation condition, the fixation point is surrounded by a signal that anticipates the task. After 1000 ms, a picture of a happy man (AM25HAS) appears for 3 s, surrounded by a green frame. In the lower example (i.e., the no preparation condition), the target, an angry woman (AF25ANS) surrounded by a purple frame, is not preceded by any signal. The color of the frame indicates the demand to perform, which is to identify either the gender or the emotion of the face, depending on the counterbalancing.

To prevent any biases, the color associated to each task was counterbalanced across participants as follows: for half of the sample the green color was associated with the gender task and the purple color was associated with the emotion task; the opposite was true for the other half of the sample. To respond, participants had to press the “Z,” “M,” “X,” or “N” keys in a QUERTY keyboard. The correspondence between key and response was also counterbalanced across participants. Specifically, for half of the sample the “Z” key was associated with “male,” “M” with “female,” “N” with “happiness,” and “X” with “anger,” while for the other half of the sample “Z” was associated with “female,” “M” with “male,” “N” with “anger,” and “X” with “happiness.” The total duration of each trial was 4 s. **Figure 1** illustrates the sequence of events in two trials.

The experimental task was programmed using E-prime software (Schneider et al., 2002). It comprised 1 block of practice (32 trials) and 8 blocks of 64 trials each, with a total duration of 40–45 min.

#### Design

The data were analyzed using SPSS 21.0 statistical software, with a 2 (Group; High Trait Cheerfulness vs. Low Trait Cheerfulness) × 2 (Task; Emotion vs. Gender) × 3 (Repetition; Complete Alternation vs. Complete Repetition vs. Partial Repetition) × 2 (Task Change; Different vs. Same) × 2 (Preparation; Preparation vs. No Preparation) mixed factorial design. The first variable was manipulated between groups, and the rest were manipulated within participants. The dependent variables were reaction time (RT), which was calculated only for correct responses also preceded by correct responses, and error percentage (EP).

### Results

Descriptive statistics are shown on **Table 1**. The analysis revealed a main effect of each of the within-participant variables: Task, *F*(1,46) = 39.56, *p* < 0.001, η^2^ = 0.46, Repetition, *F*(2,92) = 16.31, *p* < 0.001, η^2^ = 0.26, and Preparation, *F*(1,46) = 339.00, *p* < 0.001, η^2^ = 0.88. Participants were faster to respond when the task was gender identification (898 ms vs. 966 ms), when all the attributes were repeated in two consecutive trials, compared to when none were repeated or only some of them were (912 ms vs. 945 ms vs. 939 ms, respectively), and when a cue was presented anticipating the task to perform (824 ms vs. 1040 ms). Moreover, our task replicated the expected task-switching cost results, *F*(1,46) = 191.31, *p* < 0.001, η^2^ = 0.81, meaning that participants were faster when the task was repeated between two consecutive trials (134 ms task-switching cost). Additionally, as expected, this effect was modulated by attribute repetition, *F*(2,92) = 21.66, *p* < 0.001, η^2^ = 0.32, preparation conditions, *F*(1,46) = 46.82, *p* < 0.001, η^2^ = 0.50, and task type, *F*(1,46) = 10.64, *p* = 0.002, η^2^ = 0.19. Specifically, participants showed a lower task-switching cost when none of the stimulus attributes (i.e., gender or emotion) were repeated in consecutive trials, compared to when they were repeated, which generated the highest task-switching cost (101 ms vs. 182 ms). In addition, the task-switching cost was lower when the task involved recognizing the gender than when it required recognizing the emotion (116 ms vs. 154 ms), and in the preparation conditions compared to those in which there was no preparation cue (103 ms vs. 167 ms).

**Table 1:** Mean reaction time (in ms), error percentage and error standard deviation in each of the experimental conditions as a function of trait cheerfulness.

		Complete alternation				Complete repetition				Partial repetition			
		Different		Same		Different		Same		Different		Same	
		P	NP	P	NP	P	NP	P	NP	P	NP	P	NP
High Trait	Emotion	889	1225	818	1033	946	1184	751	952	930	1204	824	997
Cheerfulness		(5.69,6.53)	(6.75,7.31)	(2.54, 4.04)	(5.89,5.78)	(4.72,5.17)	(7.55,7.42)	(2.29,3.85)	(1.27,2.56)	(5.66,5.96)	(5.93,5.64)	(4.38,4.42)	(5.62,3.74)
	Gender	881	1131	786	1009	863	1098	729	869	831	1108	764	968
		(6.13,6.36)	(8.59,9.52)	(2.38,3.71)	(5.95,6.39)	(1.88,4.02)	(6.17,7.93)	(0.56,1.90)	(2.70,6.34)	(3.57,5.23)	(9.09,6.82)	(3.37,4.26)	(4.55,4.85)
Low Trait	Emotion	877	1136	820	999	950	1118	774	914	914	1142	824	964
Cheerfulness		(3.14,4.39)	(6.10,7.94)	(1.44,3.50)	(2.44,3.80)	(4.50,4.99)	(4.36,4.87)	(1.34,2.72)	(0.52,1.76)	(4.24,4.99)	(4.63,4.02)	(2.36,2.93)	(2.95,2.17)
	Gender	789	1036	730	961	817	1050	701	878	825	1045	758	930
		(3.19,4.46)	(5.29,5.17)	(1.82,3.78)	(4.77,5.00)	(2.52,3.55)	(2.55,3.86)	(1.40,2.87)	(1.07,2.50)	(3.03,4.12)	(3.28,4.35)	(3.59,4.49)	(4.29,4.54)

*P, preparation; NP, no preparation.*

**FIGURE 2:**
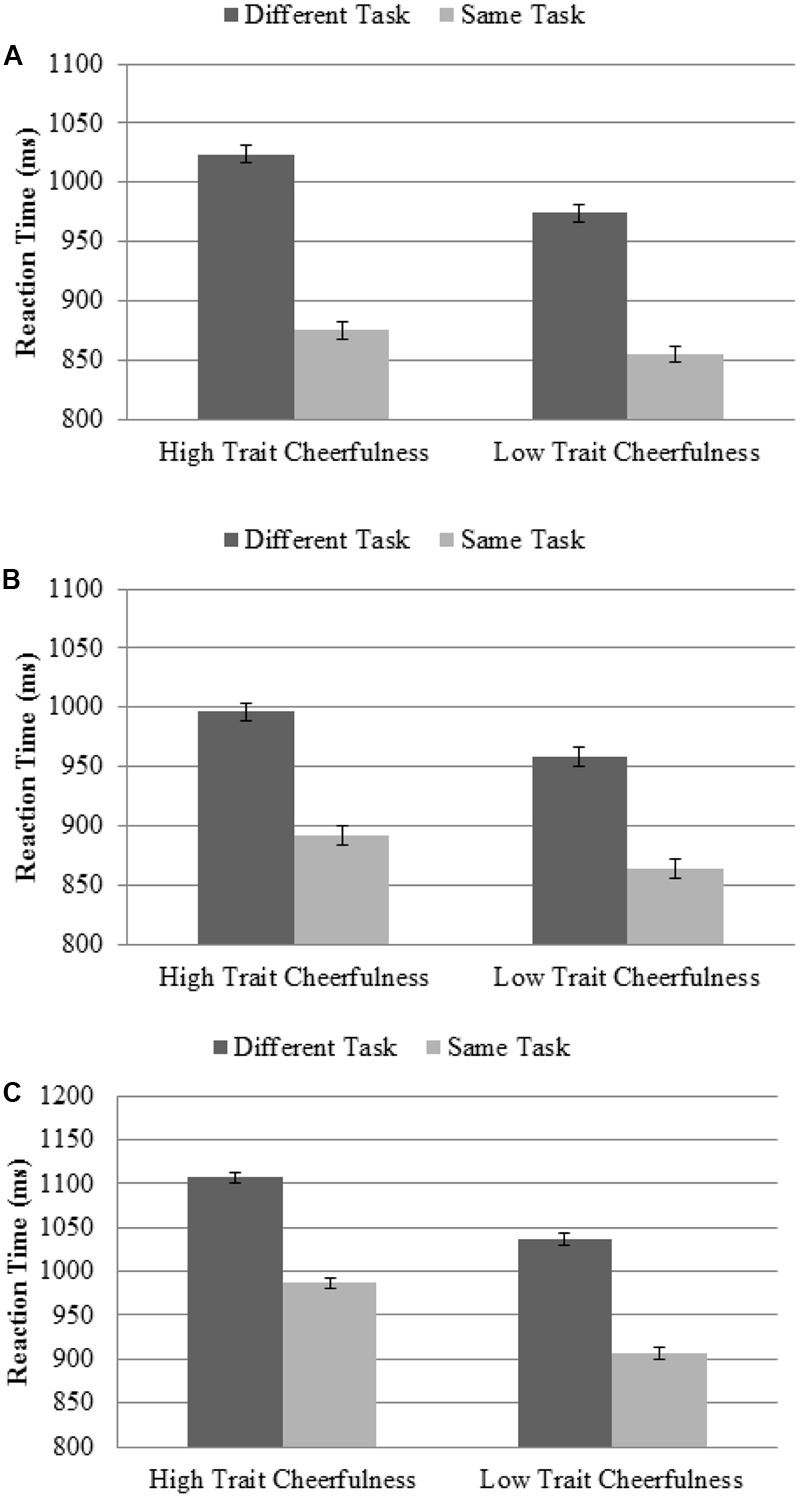
Effect of the task-switching cost as a function of trait cheerfulness group for: **(A)** Study 1; **(B)** Study 2, preparation part; and **(C)** Study 2, no preparation part. Note the lack of differences between both groups. If anything, the general trend is in the opposite direction, as individuals with high trait cheerfulness showed a higher task-switching cost (measured as the difference between a task being repeated or not) than individuals with low trait cheerfulness. The error bars represent the standard error of the mean, with variability between participants removed by means of Coussineau’s method.

More directly related to our main goal, and perhaps most importantly, we did not find any evidence of a lower task-switching cost in the high trait cheerfulness group (see **Figure 2**). In fact, we observed a non-significant trend in RT, *F*(1,46) = 2.23, *p* = 0.14, η^2^ = 0.05, in the opposite direction (149 ms task-switching cost in the high trait cheerfulness group, compared to 120 ms cost in the low trait cheerfulness group).

**FIGURE 3:**
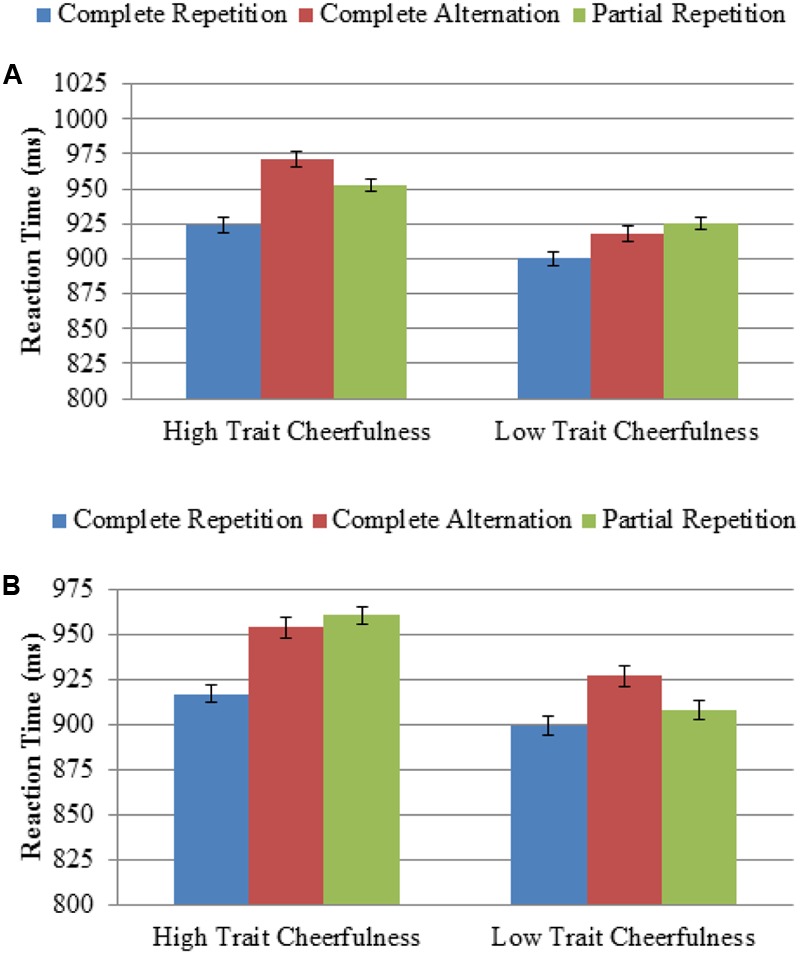
Effect of attribute repetition as a function of the trait cheerfulness group for: **(A)** Study 1; and **(B)** Study 2, preparation part. Both graphs reveal that participants with high trait cheerfulness showed a higher repetition effect than those with low trait cheerfulness, measured as an increased difference when all attributes were repeated compared to no repetition or partial repetition. The error bars represent the standard error of the mean, with variability between participants removed by means of Coussineau’s method.

Interestingly, however, group was found to modulate other relevant variables. For example, the Group × Repetition interaction was significant, *F*(2,92) = 3.30, *p* = 0.041, η^2^ = 0.07. Specifically, the previously described effect of repetition (i.e., faster responses when all attributes were repeated than when none were repeated) was present to a greater extent in the high trait cheerfulness group compared to the low trait cheerfulness group (47 ms vs. 18 ms; see **Figure 3**). The Group × Task × Preparation interaction was also significant, *F*(1,46) = 7.54, *p* = 0.009, η^2^ = 0.14, showing a higher preparation effect in the high versus low trait cheerfulness group, although this was only observed in the emotion recognition task [*F*(1,46) = 5.31, *p* = 0.026, η^2^ = 0.10, 239 ms vs. 185 ms] and not in the gender recognition task (*F* < 1).

The analysis of EP showed significant main effects in the variables Repetition, *F*(2,92) = 14.32, *p* < 0.001, η^2^ = 0.24, and Preparation, *F*(1,46) = 17.68, *p* < 0.001, η^2^ = 0.28. Overall, the pattern was very similar to that observed in RT: participants made fewer errors when the stimuli attributes were repeated than when they were not repeated or were only partially repeated (2.8% vs. 4.5% vs. 4.4%), and also when a cue was provided anticipating the demand to perform (3.2% vs. 4.7%). Again, our task replicated the predicted effects of task-switching cost, *F*(1,46) = 42.23, *p* < 0.001, η^2^ = 0.48: participants made fewer errors when the task was repeated in two consecutive trials (2% task-switching cost). Furthermore, as expected, this effect was significantly modulated by attribute repetition, *F*(2,92) = 4.35, *p* = 0.016, η^2^ = 0.09, and marginally modulated by task type, *F*(1,46) = 3.36, *p* = 0.073, η^2^ = 0.07. Specifically, we observed a higher task-switching cost when all attributes were repeated than when no attributes were repeated or when only some were repeated (2.9% vs. 2.2% vs. 1%). We also observed a trend toward a higher cost when the task to perform was expressed emotion recognition (2.5% vs. 1.6%).

Regarding our main goal, the analysis revealed a main effect of Group (1,46) = 6.80, *p* = 0.012, η^2^ = 0.13, which reflected that individuals with high trait cheerfulness had a higher EP than those with low trait cheerfulness (4.7% vs. 3.1%). We also observed a significant interaction between Group × Task × Task Change, *F*(1,46) = 5.52, *p* = 0.023, η^2^ = 0.11. The interaction revealed that individuals with high trait cheerfulness showed a higher task-switching cost than those with low trait cheerfulness, although this only applied to the gender recognition task (2.6% vs. 0.5%), not to the emotion recognition task (2.4% vs. 2.7%).

**FIGURE 4:**
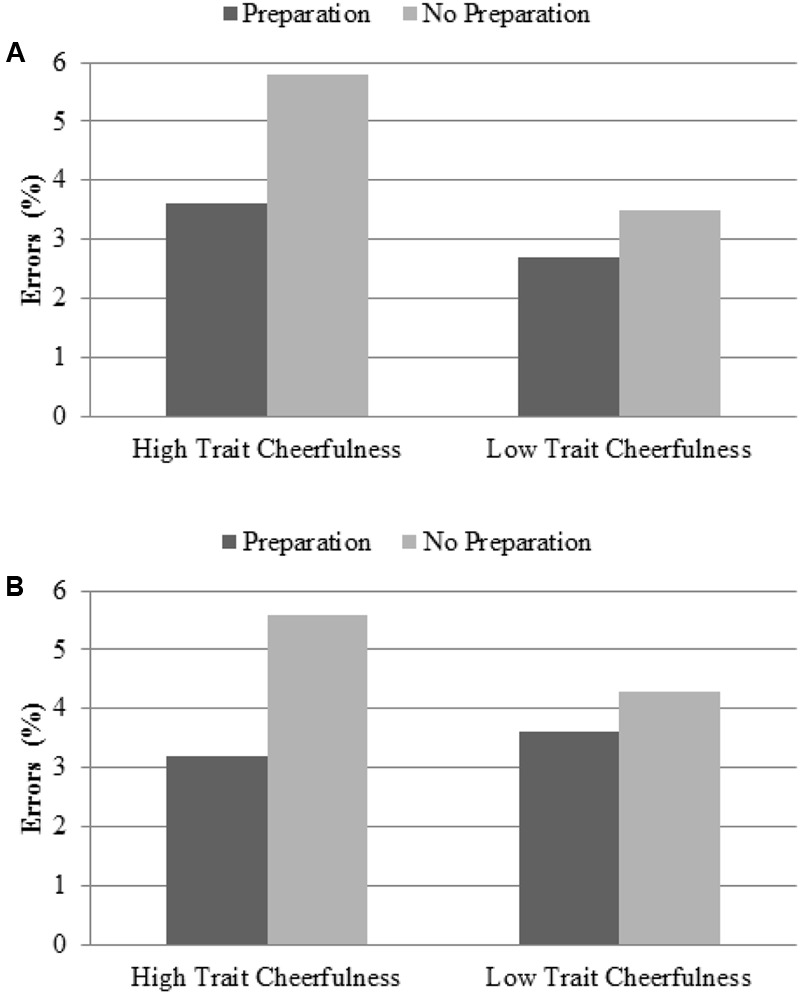
Effect of preparation as a function of the trait cheerfulness group for: **(A)** Study 1; and **(B)** Study 2, preparation part. Both graphs reveal that participants with high trait cheerfulness showed a higher effect of preparation than those with low trait cheerfulness, measured as a greater reduction in error percentage when the target was preceded by a signal that anticipated the upcoming demand than when it was not present.

Additionally, a higher effect of preparation was observed in individuals with high versus low trait cheerfulness (2.2% vs. 0.8%) regardless of the task, as reflected by the marginally significant Group × Preparation interaction, *F*(1,46) = 3.90, *p* = 0.054, η^2^ = 0.08 (see **Figure 4**).

### Discussion

In this study, our aim was to replicate the modulation of cognitive flexibility by trait cheerfulness observed in a previous study and further analyze these relationships. Results proved that the task-switching paradigm we used was an efficient instrument to study this process, since the usual task-switching cost pattern was observed (e.g., modulation by task type, attribute repetition, and preparation cue). However, it is important to note that, overall, our data reflected that individuals with high trait cheerfulness do not seem to show a lower task-switching cost than those with low trait cheerfulness. If anything, the little evidence collected indicated the opposite, as the EP results revealed a higher, not lower, task-switching cost in individuals with high trait cheerfulness in the gender recognition task. The pattern observed in RT followed the same trend, although differences were not significant. Hence, our result pattern did not support the idea of a link between trait cheerfulness and a lower task-switching cost and thus increased cognitive flexibility.

However, we did observe significant effects of group with regard to the repetition of the stimuli attributes and the prior preparation to them. Specifically, individuals with higher trait cheerfulness showed a larger effect of stimuli repetition and a larger effect of task preparation, particularly in the expressed emotion recognition task. We consequently decided to carry out a second study with the goal of verifying if, indeed, trait cheerfulness did not modulate the task-switching cost, and also of exploring whether the effects of repetition and preparation were consistent.

## Study 2

Considering the findings of Study 1, we conducted Study 2 to further explore whether trait cheerfulness modulated the task-switching cost, and studying whether it was possible to replicate the modulation by trait cheerfulness of the repetition of the stimuli attributes and the preparation to the stimuli. A previous study had produced some evidence suggesting that individuals with high trait cheerfulness show a lower task-switching cost compared to individuals with low trait cheerfulness (López-Benítez et al., unpublished). Yet, this effect was not replicated in Study 1. This could be due to the presence of a demand anticipating cue in half of the trials, given that, if the participant has sufficient preparation, the effect of task-switching cost as a function of trait cheerfulness may diminish or even disappear. Note that, in the previous study, no preparation cue was presented.

Therefore, the present study had two parts (of four blocks each) that were counterbalanced. Half of the blocks followed the same structure as in Study 1, but in the other half the demand anticipating cue was eliminated (as in López-Benítez et al., unpublished). If the determining factor in the differential effect of task-switching cost as a function of trait cheerfulness is anticipation of the demand, we hypothesized that participants with high versus low trait cheerfulness will show a lower task-switching cost (i.e., higher cognitive flexibility) in an experiment in which the demand is not anticipated. Furthermore, in line with Study 1, we expected to find a higher effect of both attribute repetition and preparation to the task in individuals with high trait cheerfulness than in those with low trait cheerfulness.

### Material and Methods

#### Participants

Following the same method as in Study 1, 48 new students from the University of Granada were selected out of 569 people^2^. In this case, the high trait cheerfulness group was made up of 25 participants (19 women, mean age 22.36 years, *SD* = 4.37, cut-off score ≥ 3.50), while the low trait cheerfulness group comprised 23 participants (19 women, mean age 21.83 years, *SD* = 3.42, cut-off score ≤ 2.63). All the participants had normal or corrected-to-normal vision, performed the task voluntarily, and received course credit in exchange for participating. They signed an informed consent and had the possibility to stop the experimental session without any consequences. Data from one participant were not taken into account because the number of correct responses was low compared to the group (more than 2.5 SD below the group mean). Again, the study was part of a broader research project, approved by the Ethics Committee of the University of Granada, in accordance with the 1964 Declaration of Helsinki.

#### Stimuli and Procedure

The stimuli and procedure were the same as in Study 1, with two exceptions. First, instead of being composed of eight similar blocks, the study was divided into two distinct parts, each of which comprised four blocks. The first part was the same as in Study 1, but in the second part no pre-target cue was given to indicate the upcoming task. Both parts were counterbalanced between groups. Second, in order to maintain the alertness level of participants, an audio feedback signal was used every time a wrong response or no response was given.

#### Design

The data were analyzed using SPSS 21.0 statistical software. We decided to analyze this study separately depending on whether the trials with a previous preparation condition were mixed with those that did not have any (preparation part), or there was rather no mix between trials (no preparation part). We used the same design as in Study 1 in the blocks in which there was a possibility of preparing for the demand: 2 (Group; High Trait Cheerfulness vs. Low Trait Cheerfulness) × 2 (Task; Emotion vs. Gender) × 3 (Repetition; Complete Alternation vs. Complete Repetition vs. Partial Repetition) × 2 (Task Change; Different vs. Same) × 2 (Preparation; Preparation vs. No Preparation). The same design was used for the analysis of the blocks of trials in which there was no possibility of preparing for the demand, with the sole exclusion of the preparation variable: 2 (Group; High Trait Cheerfulness vs. Low Trait Cheerfulness) × 2 (Task; Emotion vs. Gender) × 3 (Repetition; Complete Alternation vs. Complete Repetition vs. Partial Repetition) × 2 (Task Change; Different vs. Same). Again, RT, which was calculated only for correct responses that were also preceded by correct responses, and EP were analyzed as dependent variables.

### Results

#### Analysis of the Preparation Part

Descriptive statistics are shown on **Table 2**. The analysis revealed a main effect of each of the within-participant variables: Task, *F*(1,45) = 52.53, *p* < 0.001, η^2^ = 0.54, Repetition, *F*(2,90) = 13.51, *p* < 0.001, η^2^ = 0.23, and Preparation, *F*(1,45) = 261.45, *p* < 0.001, η^2^ = 0.85. As in Study 1, participants were faster to respond when the task was gender recognition (877 ms vs. 978 ms), when all the attributes were repeated between two consecutive trials, compared to no or partial attribute repetition (908 ms vs. 940 ms vs. 934 ms), and when a cue was used to anticipate the demand (810 ms vs. 1044 ms). Once again, our procedure additionally showed the expected task-switching cost effects, *F*(1,45) = 74.24, *p* < 0.001, η^2^ = 0.62, meaning that participants’ responses were faster when the task was repeated in two consecutive trials (99 ms task-switching cost). This effect was modulated by attribute repetition, *F*(2,90) = 24.66, *p* < 0.001, η^2^ = 0.35, preparation conditions, *F*(1,45) = 30.17, *p* < 0.001, η^2^ = 0.40, and task type, *F*(1,45) = 9.71, *p* = 0.003, η^2^ = 0.18. Thus, the task-switching cost was lower when none of the stimuli attributes (i.e., gender or emotion) were repeated in consecutive trials than when they were repeated; the latter condition generated the highest task-switching cost (46 ms vs. 166 ms). The task-switching cost was also lower in the preparation conditions (63 ms vs. 135 ms) and when the task was gender recognition (75 ms vs. 127 ms). In addition, the lower task-switching cost in preparation conditions was modulated by attribute repetition, *F*(2,90) = 8.34, *p* < 0.001, η^2^ = 0.16, as this effect was lower when only some or none of the stimuli attributes were repeated between two consecutive trials than when all the attributes were repeated (27 ms vs. 49 ms vs. 145 ms).

**Table 2:** Mean reaction time (in ms), error percentage, and error standard deviation in each of the experimental conditions as a function of trait cheerfulness.

		Complete alternation						Complete repetition						Partial repetition					
		Different			Same			Different			Same			Different			Same		
		P	NP	NPP	P	NP	NPP	P	NP	NPP	P	NP	NPP	P	NP	NPP	P	NP	PNP
High Trait	Emotion	915	1219	1123	804	1063	1059	891	1174	1178	804	937	931	936	1187	1148	847	1081	1034
Cheerfulness		(5.00, 7.43)	(8.78, 10.08)	(5.80, 7.50)	(1.64, 4.63)	(4.21, 7.86)	(4.11, 7.75)	(8.82, 10.10)	(6.97, 16.63)	(5.12, 6.83)	(1.90, 5.28)	(0.97, 3.42)	(1.99, 3.70)	(5.24, 5.77)	(8.20, 11.13)	(5.07, 6.67)	(4.36, 5.75)	(6.92, 6.33)	(4.23, 5.11)
	Gender	756 (3.13, 6.02)	1056 (4.15, 9.97)	1057 (3.53, 4.50)	785 (2.00, 4.20)	1032 (7.30, 11.03)	1008 (4.94, 6.31)	819 (3.13, 6.02)	1089 (4.79, 9.91)	1062 (4.80, 5.82)	752 (1.44, 4.00)	869 (1.74, 5.02)	909 (2.09, 4.48)	811 (2.90, 5.46)	1099 (4.86, 5.97)	1073 (5.76, 7.71)	736 (3.30, 4.94)	989 (3.69, 5.88)	978 (5.21, 6.03)
Low Trait	Emotion	886 (4.17, 8.74)	1142 (6.39, 8.74)	1098 (8.51, 9.00)	860 (3.92, 9.16)	1049 (4.14, 8.80)	968 (4.44, 6.24)	915 (5.48, 6.74)	1180 (7.27, 9.72)	1078 (5.62, 7.17)	790 (2.13, 6.49)	926 (1.28, 3.30)	876 (1.06, 3.47)	904 (5.57, 6.13)	1129 (5.94, 6.66)	1109 (6.14, 7.25)	831 (4.46, 6.00)	1009 (7.50, 9.36)	984 (3.51, 4.73)
Cheerfulness	Gender	732 (2.62, 7.39)	1002 (2.18, 4.94)	964 (4.33, 5.47)	750 (2.35, 5.29)	991 (3.56, 8.07)	898 (4.01, 4.42)	790 (3.89, 7.98)	1072 (3.16, 6.59)	988 (4.98, 6.96)	691 (3.31, 8.35)	827 (1.82, 5.88)	824 (2.91, 4.69)	746 (3.00, 6.28)	996 (4.73, 6.05)	978 (4.70, 5.13)	701 (2.30, 4.12)	947 (3.28, 5.06)	892 (4.10, 6.47)

*P, preparation; NP, no preparation; NPP, no preparation part.*

Regarding our goal, and as shown in **Figure 2**, no evidence was found of a lower task-switching cost in individuals with high versus low trait cheerfulness (*F* < 1). However, we replicated the modulation of attribute repetition by trait cheerfulness, as reflected in the Group × Repetition interaction, *F*(2,92) = 3.30, *p* = 0.041, η^2^ = 0.07. This confirmed that, compared to individuals with low trait cheerfulness, those with high trait cheerfulness showed a higher effect of repetition when all the attributes were repeated between two consecutive trials than when only some of them were repeated (44 ms vs. 10 ms; see **Figure 3**).

Error percentage analysis revealed significant main effects in the following variables: Task, *F*(1,45) = 10.86, *p* = 0.002, η^2^ = 0.19, Repetition, *F*(2,90) = 3.13, *p* = 0.049, η^2^ = 0.07, and Preparation, *F*(1,45) = 11.57, *p* = 0.001, η^2^ = 0.20. In general, the pattern was very similar to that observed in RT and with that observed in Study 1. In fact, participants made fewer errors when the task was gender recognition (3.3% vs. 5%), when the stimuli attributes were repeated, compared to no repetition or partial repetition (3.5% vs. 4.3% vs. 4.8%), and when a cue was given anticipating the demand (3.4% vs. 4.9%). Once more, we observed the expected effects of task-switching cost, *F*(1,45) = 14.90, *p* < 0.001, η^2^ = 0.25, reflected in a higher accuracy when the task was repeated in two consecutive trials (1.5% task-switching cost). Additionally, and as expected, this effect was significantly modulated by attribute repetition, *F*(2,90) = 4.91, *p* = 0.010, η^2^ = 0.10, and by task type, *F*(1,45) = 5.83, *p* = 0.020, η^2^ = 0.12. In this regard, we found that the task-switching cost was higher when all the attributes were repeated, compared to no repetition or partial repetition (3.3% vs. 0.6% vs. 0.6%), and when the task was expressed emotion recognition (2.4% vs. 0.5%).

Regarding our main goal, no evidence was found that trait cheerfulness modulated the effect of task-switching cost (*F* < 1). However, as observed in Study 1, the Group × Preparation interaction was found to be marginally significant, *F*(1,45) = 3.70, *p* = 0.061, η^2^ = 0.08, replicating the trend toward a higher overall effect of preparation in participants with high versus low trait cheerfulness (2.4% vs. 0.7%; see **Figure 4**).

### Analysis of the No Preparation Part

Descriptive statistics are shown on **Table 2**. As in the previous studies, the analysis revealed a main effect of each of the within-participant variables: Task, *F*(1,45) = 22.95, *p* < 0.001, η^2^ = 0.34, and Repetition, *F*(2,90) = 17.59, *p* < 0.001, η^2^ = 0.28. Specifically, participants were faster when the task was gender recognition (969 ms vs. 1049 ms) and when all attributes between two consecutive trials were repeated, as opposed to no repetition or partial repetition of attributes (981 ms vs. 1022 ms vs. 1025 ms). Once again, our study showed that participants were faster when the task was repeated between two consecutive trials (124 ms task-switching cost), *F*(1,45) = 185.69, *p* < 0.001, η^2^ = 0.81. As expected, this effect was again modulated by task type, *F*(1,45) = 12.60, *p* = 0.001, η^2^ = 0.22, and attribute repetition, *F*(2,90) = 28.72, *p* < 0.001, η^2^ = 0.39. In this regard, the task-switching cost was lower when the task was gender recognition (101 ms vs. 148 ms) and also when none of the stimuli attributes (i.e., gender or emotion) were repeated in consecutive trials, compared to when they were repeated, which generated the highest task-switching cost (78 ms vs. 192 ms).

With regard to our main goal, as can be seen in **Figure 2**, individuals with high trait cheerfulness did not show a lower task-switching cost than those with low trait cheerfulness (*F* < 1). In fact, cheerfulness did not modulate any other variable, such as repetition (*F* < 1).

The accuracy analysis revealed a main effect of the Repetition variable, *F*(2,90) = 5.13, *p* = 0.008, η^2^ = 0.10, that is, participants made fewer errors when all the stimuli attributes were repeated between two trials than when none were repeated (3.6% vs. 5%). As expected, accuracy increased when the task was repeated in two consecutive trials, *F*(1,45) = 23.85, *p* < 0.001, η^2^ = 0.35, showing a 1.8% task-switching cost. This effect was also modulated by task type, *F*(1,45) = 9.11, *p* = 0.004, η^2^ = 0.17, and marginally modulated by attribute repetition, *F*(2,90) = 2.68, *p* = 0.074, η^2^ = 0.06. In other words, the task-switching cost was lower when the task was gender recognition (3.6% vs. 5%) and also when no (or only some) attributes were repeated, compared to complete attribute repetition (1.1% vs. 1.1% vs. 3.1%).

As happened with RT, individuals with high trait cheerfulness did not show a lower task-switching cost than individuals with low trait cheerfulness (*F* < 1). We did not find any relationship with other relevant variables either (*F* < 1).

### Discussion

The goal of this study was to study whether individuals with high trait cheerfulness showed a lower task-switching cost by exploring whether this modulation could be caused by the presentation of a cue anticipating the demand and hence the response. We also intended to verify whether the higher effect of attribute repetition and task preparation in participants with high trait cheerfulness found in Study 1 was replicated.

As in Study 1, Study 2 confirmed the suitability of the task for the study of task-switching cost. Again, our data did not provide evidence that individuals with higher trait cheerfulness showed higher cognitive flexibility, measured as a lower task-switching cost, than those with low trait cheerfulness.

However, although only in the preparation part, individuals with high trait cheerfulness again displayed both a larger effect of attribute repetition between two consecutive trials, and a larger effect of task preparation, thus replicating the findings of Study 1.

## General Discussion

The main aim of this research was to study the modulation of cognitive flexibility processes by trait cheerfulness, as a temperamental basis of sense of humor (Ruch et al., 1996, 1997), by using a task-switching paradigm. Although the procedure used showed the typical effects of task-switching cost, the results reflected that high trait cheerfulness people did not show a lower task-switching cost, that is, a better cognitive flexibility compared to low trait cheerfulness individuals.

Some authors have pointed out the potential benefits of positive emotions in areas such as cognition (see, for example, Lyubomirsky et al., 2005, for a review). Specifically, it has been observed that positive affect reduces the task-switching cost in a paradigm with no emotional implications (i.e., task-switching between color and shape; Yang and Yang, 2014). It has also shown that some personality characteristics may benefit (DeYoung et al., 2005) or impair (Compton, 2000; Campbell et al., 2011) performance on cognitive flexibility tasks. Additionally, previous research has suggested that cognitive flexibility processes could be involved in contexts where people have to detect or enjoy humor (Polimeni et al., 2010; Weiss et al., 2013) as well as in situations in which emotion regulation strategies are applied to alter an event’s affective impact on people’s affective state (Malooly et al., 2013; Gul and Khan, 2014). Therefore, considering that trait cheerfulness is a positive predisposition to detect, produce, enjoy, and maintain humoristic stimuli as well as positive emotions (Ruch and Hofmann, 2012), it has been positively associated with personality variables related to a better cognitive flexibility (Carretero-Dios et al., 2014), and with a better coping with negative emotions (Papousek and Schulter, 2010). On the other hand, considering the results of our previous study, it could then be inferred that individuals with high trait cheerfulness should have a lower task-switching cost, that is, a higher cognitive flexibility, compared to individuals with low trait cheerfulness. Our findings, however, did not confirm this hypothesis.

From a personality perspective, our results could be partially explained. On the one hand, it is true that trait cheerfulness is closely related to extraversion (Ruch and Köhler, 2007; Carretero-Dios et al., 2014), which is negatively associated with the performance in tasks that involve cognitive flexibility (Campbell et al., 2011). This fact could justify that individuals characterized by high trait cheerfulness did not show higher cognitive flexibility in our study (as measured by a lower task-switching cost). If anything, our results indicated the opposite trend, i.e., a higher task-switching cost for high trait cheerfulness people. Moreover, trait cheerfulness is also positively linked to openness and agreeableness, and negatively related to neuroticism (Carretero-Dios et al., 2014), which promote (Jensen-Campbell et al., 2002; DeYoung et al., 2005) and impair (Compton, 2000), respectively, cognitive flexibility. In this sense, high trait cheerfulness people should have a greater ability to shift their mental set when they are working on different tasks. However, this is not the case.

In addition, it has been sometimes reported that positive states do not have benefits on cognitive processes (Mitchell and Phillips, 2007). For example, some studies have failed to find a clear pattern of task-switching cost reduction when a motivational intensity induction is carried out (high interest) compared to negative emotional states or a control condition (Zhou and Siu, 2015). Others studies have not found a clear pattern of benefits from positive affective induction in multitasking conditions either (Morgan and D’Mello, 2016). Contradictory results were also observed by Phillips et al. (2002), who revealed a poorer performance after a positive affective state induction, compared with a neutral induction, in task-switching conditions between naming the color versus the word in Stroop tasks. Yet, they found a smaller difference between alternation and non-alternation conditions in a verbal fluency task (i.e., alternating or not between saying words starting with a specific letter and words from a specific semantic category).

Furthermore, it is important to note the nature of the task and how cognitive flexibility is measured. In our studies, flexibility is assessed as the ability to change between mental sets for adapting to new demands in a cognitive task. In this sense, it might be possible that the cognitive nature of this task involves cognitive flexibility processes different from those that are relevant to recognize humor (Weiss et al., 2013), which would be more associated to cheerfulness. In addition, trait cheerfulness is related to a greater coping with and recovery from negative emotions (see Papousek and Schulter, 2010; Ruch and Hofmann, 2012), which has also been associated with cognitive flexibility processes (Malooly et al., 2013). In a recent study (López-Benítez et al., under review), it has been found that people with high versus low trait cheerfulness frequently use reappraisal strategies in their daily lives. However, they did not have a better ability to apply reappraisal strategies for down- regulating negative emotions. In this sense, if our task is testing cognitive flexibility as ability rather than a general use of it, it could be thought that the frequent use of reappraisal strategies is not enough to also have a greater cognitive flexibility. In any case, future studies are needed to test these hypotheses.

The present pattern of results could be influenced by the sample size. To solve this limitation, we carried out an omnibus analysis with data from Study 1 (*N* = 49), Study 2 (*N* = 48), and our unpublished study (*N* = 46), with a total of 72 high trait cheerfulness participants and 71 low trait cheerfulness participants. Only trials that appeared in all studies were selected, that is, trials where there was not a prior preparation to the task, given that the preparation condition was presented in some studies but not in others. The mixed ANOVA with Task Change (Different vs. Same) as within participants variable and Group (High Trait Cheerfulness vs. Low Trait Cheerfulness) and Study as between participants variables showed a complete absence of Group × Task Change interaction, *F*(1,137) = 0.07, *p* = 0.791, η^2^ = 0.00.

In order to see whether this absence of evidence could be taken as evidence for absence of modulation of group over the task-switching costs, a Bayesian approach was used. This procedure assesses how much support we could obtain for the null hypothesis through the Bayes Factor (BF_10_), which represents how strongly a result supports our hypothesis (i.e., lower task-switching cost for high trait cheerfulness people, or Group × Task Change interaction, H_1_) over the null hypothesis (i.e., no Group × Task Change interaction). Three ranges of values for BF_10_ are commonly accepted to interpret the output: (a) evidence of the absence of an effect (from 0 up to 0.33); (b) inconclusive evidence (from 0.33 up to 3); (c) evidence of an effect (from 3 and up). The Bayesian analysis was carried out with JASP Team (2017). Our results indicated, again reflecting that task-switching cost did not depend on trait cheerfulness, substantial evidence for a null effect (BF_10_ = 0.186, for the Group × Task Change interaction or a *t*-test comparing the two groups on the task-switching cost).

A tentative explanation of the present results is related to the subject of this research and the demands required by the task itself. Cheerfulness is a positive affective predisposition associated with sense of humor (Ruch and Köhler, 2007). It is therefore related to the manifestation, enhancement, and maintenance of positive emotions, along with a lower manifestation of negative emotions and a higher resilience to them (Zweyer et al., 2004; Papousek and Schulter, 2010). This endows it with qualities that are very closely linked to processes of an emotional nature, such as induction processes, regulation, and emotional intelligence (e.g., Ruch, 1997; Yip and Martin, 2006), and processes more related to social interaction and empathy (e.g., Ruch and Köhler, 2007; Beermann and Ruch, 2009). From this viewpoint, given the affective, humoristic, communicative, expressive, and social characteristics that compound trait cheerfulness, it might be possible that trait cheerfulness has a higher predictive power and play a relevant role in tasks that involve processes of this nature, compared to cognitive tasks which do not include elements typical of humoristic, emotional, or social stimulation. Further research needs to be carried out in this field to clarify these ideas.

In addition, and although this was not our main goal, in Study 1 and in the preparation part of Study 2 we observed that, compared to individuals with low trait cheerfulness, those with high trait cheerfulness showed a higher effect of attribute repetition between two consecutive trials (e.g., Hommel, 2004). They also showed a tendency toward a higher effect of preparation when presented with a cue anticipating the demand in a trial that immediately followed (e.g., Kiesel et al., 2010) that was even higher in the expressed emotion recognition task (Study 1).

To our knowledge, no studies have explored the modulation of the effects of attribute repetition by predisposition to affective states (or affective states themselves). However, if our findings are confirmed, it may be possible to explain them in terms of the broaden-and-build theory (Fredrickson, 2001). According to this approach, positive states often lead to a more holistic processing of the context, thus expanding the attention focus (see, for example, Johnson et al., 2010). Taking into account that trait cheerfulness is a predisposition toward positive affective states, it could be inferred that individuals with high trait cheerfulness are defined by a more global processing style. In this sense, even if all participants were to benefit from attribute repetition between consecutive trials and from a cue anticipating the next demand, it would be possible to theorize that, due to their more global mindset configuration, individuals with high trait cheerfulness benefit more from these facilitation effects, having the information on the demand to carry out more active in their short-term memory, which would improve their immediate response, particularly in the expressed emotion recognition task (Study 1), which is considered more complicated (e.g., Egner et al., 2008; Ochsner et al., 2009).

Another possible explanation is derived from affective induction contexts. In a recent study, López-Benítez et al. (in press) have found that, compared to low trait cheerfulness people, individuals characterized by high trait cheerfulness experienced a larger affective state change as a consequence of watching amusing and sad stimuli. The authors interpreted this finding as larger affective sensitivity or permeability to the environment, thus promoting some psychological, social, and physical benefits in high trait cheerfulness individuals (e.g., Yip and Martin, 2006; Carretero-Dios et al., 2014; Delgado-Domínguez et al., 2016). In this sense, it is possible that the presentation of a cue to anticipate the task might be a powerful element to capture and focus high cheerfulness people’s attention, promoting a better permeability (larger preparation effects) to it. Moreover, this fact also would explain, at least partially, the larger attribute repetition effect for high trait cheerfulness individuals that was only significant when a cue that prepares to a subsequent demand was displayed.

Therefore, from this point of view, it might be possible that high trait cheerfulness individuals have a higher receptivity to process useful and relevant nuances and contextual cues, which could help them to a better adaptation to the environment. In any case, future studies should replicate and extend these findings in order to understand the role of trait cheerfulness on these phenomena.

Notwithstanding the importance of the results, our study had some limitations. First, as pointed out above, participants in our studies were selected according to their trait cheerfulness scores. Ruch et al. (1996, 1997) suggest that the temperamental basis of sense of humor have two manifestations, as traits and as states, which are closely related to one another. Clear dissociations have been observed between traits and states, which have differential modulation effects on attentional processes in other areas such as anxiety (Pacheco-Unguetti et al., 2010). In addition, and following Fredrickson’s (2001) theoretical proposal, a positive affective induction rather than a positive trait might have a greater impact in aspects such as cognitive resources. Therefore, it would be interesting to verify whether the induction of state cheerfulness, as opposed to the selection of participants with high trait cheerfulness, would have the same effects as those caused by trait cheerfulness or if, on the other hand, participants’ state at the time of the task is a more powerful predictive factor to explain cognitive flexibility. Moreover, further research is needed to assess whether other elements of sense of humor are relevant for making predictions on this type of processes. For example, taking into account that the task used here might put participants in a telic state of mind, that is, more goal oriented (Apter and Smith, 1977) and assuming that seriousness is described from a cognitive, attitudinal, and reflexive perspective, trait or, even more importantly, state seriousness may modulate to a greater extent the effect of these processes, which have a more cognitive nature. Additionally, based on studies that have found a relationship between negative affective states and a poorer performance in multitasking conditions, which require high cognitive flexibility (Morgan and D’Mello, 2016), it could be inferred that bad mood, through its affective properties, may also modulate cognitive flexibility, leading to a lower task-switching cost.

Second, taking into account that trait cheerfulness is linked to personality characteristics that may affect the performance on tasks that require cognitive flexibility (e.g., Compton, 2000; Jensen-Campbell et al., 2002), they should be incorporated in future studies together with related variables such as, for example, optimism, to observe their differential weight in cognitive tasks compared to trait cheerfulness.

Finally, assuming the conceptualization of the cheerfulness construct (for a review, see Ruch and Hofmann, 2012), it might be more interesting to analyze the modulation of emotional induction processes by cheerfulness, in its trait and state manifestation, in the presence not only of positive but also of negative emotions. It would also be interesting to explore its possible relationship with emotion regulation strategies, which are involved in these processes with the goal of modifying the affective response experienced by an individual.

In short, two studies were conducted in this research to verify whether individuals with high trait cheerfulness, compared to those with low trait cheerfulness, showed higher cognitive flexibility, manifested as a lower task-switching cost. The results did not confirm this scenario. This is important taking into account that a relation between this cheerfulness and cognitive flexibility can be predicted from the literature on both humor and cheerfulness. Nevertheless, individuals with high versus low trait cheerfulness showed higher effects of attribute repetition and task preparation. Therefore, although replication of this finding seems necessary, it suggests a new path of exploration. The higher permeability to contextual cues of high cheerfulness individuals shown in the current and previous studies (López-Benítez et al., in press) could underlay a better adaptation to the environment that calls for future research. In addition, new studies should analyze whether these effects can be generalized to other cognitive processes such as creativity while exploring the modulation of affective processes by cheerfulness.

## Author Contributions

Conceived and designed the experiments: RL-B, JL, AA, HC-D. Performed the experiments: RL-B. Analyzed the data: RL-B, JL, AA, HC-D. Interpreted the data and drafted the manuscript: RL-B, JL, AA, HC-D. All authors read and accepted the final manuscript submitted for publication.

## Conflict of Interest Statement

The authors declare that the research was conducted in the absence of any commercial or financial relationships that could be construed as a potential conflict of interest.

## Footnotes

1 Ruch proposed the term *exhilaration* or *amusement* (hilarity, joy, euphoria, or rejoicing) as a name for this emotion, which is used to denote either the process of making cheerful or the temporary rising and fading out of a cheerful state (Ruch and Köhler, 2007, p. 205).

2 The total sample of 569 people was composed by the 244 people from Study 1, and 325 new participants. From the total sample, only participants with extreme scores and who did not perform the experimental task in Study 1 were selected.
